# Prenatal diagnosis of congenital left ventricular diverticulum

**DOI:** 10.1590/0100-3984.2016.0129

**Published:** 2018

**Authors:** Lásaro André Leite Costa, Hélio Antonio Guimarães Filho, Carlos Fernando Melo Júnior, Edward Araujo Júnior

**Affiliations:** 1 Cetrim - Centro de Treinamento em Imaginologia, João Pessoa, PB, Brazil; 2 Universidade Federal da Paraíba (UFPB), João Pessoa, PB, Brazil; 3 Escola Paulista de Medicina da Universidade Federal de São Paulo (EPM-Unifesp), São Paulo, SP, Brazil

**Figure 1:**
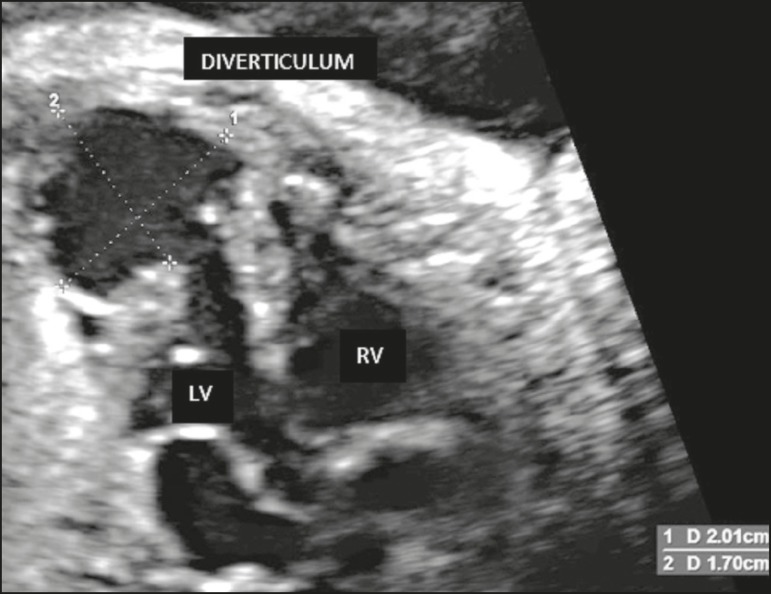
Four-chamber ultrasound view of the fetal heart, showing a diverticulum in the free wall of the left ventricle, measuring approximately 1.7 cm × 2.0 cm. LV, left ventricle; RV, right ventricle.

**Figure 2:**
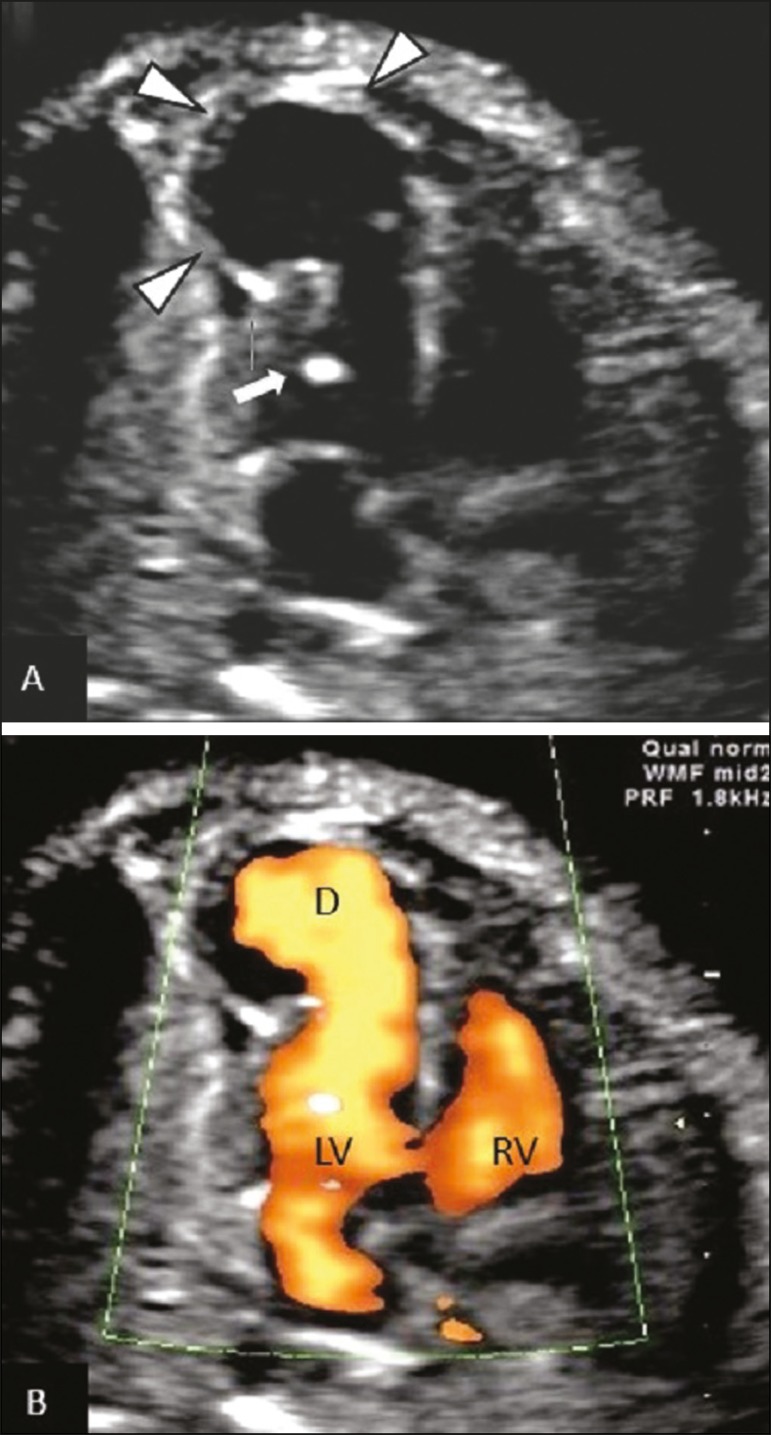
Four-chamber ultrasound view of fetal heart. **A:** Diverticulum near the apex of the left ventricle (arrowheads) and "golf ball" in the left ventricle (arrow). **B:** Power Doppler ultrasound showing the blood flow within the diverticulum during the cardiac cycle. LV, left ventricle; RV, right ventricle; D, diverticulum.

**Figure 3:**
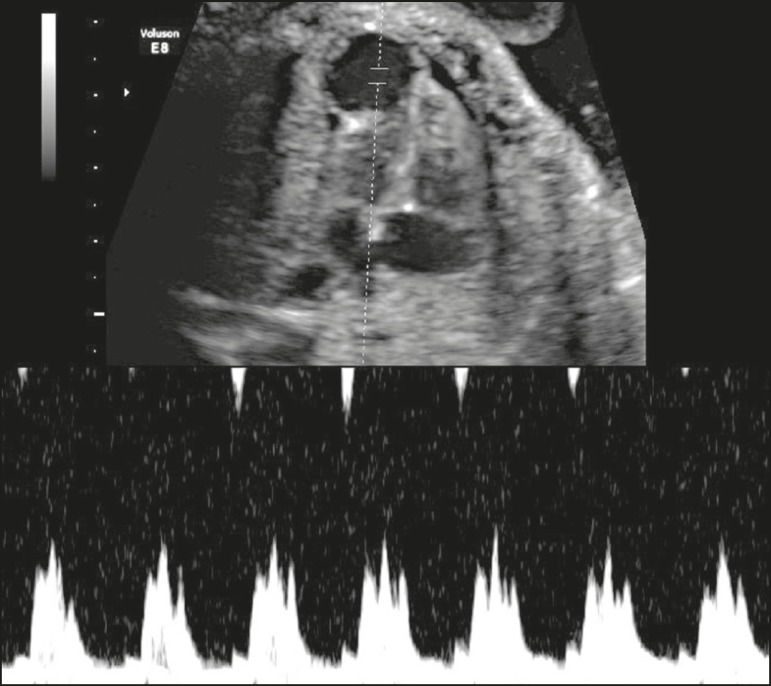
Spectral Doppler ultrasound showing triphasic flow and high pulsatility within the diverticulum.

Dear Editor,

Fetal cardiac anomalies involving the atrial septum, ventricular outflow tract, chambers, and valves are often found in routine examinations. However, prenatal detection of left ventricular diverticulum (LVD) is rare^(1,2)^. A 28-year-old primiparous pregnant woman underwent a routine ultrasound in the 22nd week. The fetal heart was found to be topic, with normal axis and volume. In the four-chamber view, we observed a structural cardiac abnormality characterized by the presence of an anechoic sac-like formation in the free wall of the left ventricle, near the apex of the heart, rounded and in the form of an exophytic cavity with thin walls, measuring approximately 1.7 cm × 2.0 cm (Figure 1). The two-dimensional examination revealed slight contractility of its walls, and a rhythm consistent with predominance ventricular rate, which would suggest a diagnosis of LVD. Power Doppler ultrasound showed filling of the entire cavity during ventricular systole and emptying during diastole (Figure 2). Spectral Doppler ultrasound showed triphasic flow and high pulsatility within the LVD (Figure 3); the cardiac morphology was otherwise normal. The remaining fetal anatomy was also normal. During prenatal care, the fetal heart showed no significant changes in its dimensions or its other aspects, and no associated complications were identified. Cesarean section was performed at 35 weeks of gestation, because of fetal distress. The newborn weighed 2183 g; the 1- and 5-minute Apgar scores were 7 and 10, respectively. Postnatal echocardiography confirmed the LVD in the free wall of the left ventricle. On the 3rd day of life, the newborn underwent surgery to correct the defect, and there were no postoperative complications. The newborn remained in the neonatal intensive care unit for 9 days and was discharged from the hospital on the 18th day of life, with preserved cardiac function and no complications.

An LVD is defined as a protrusion of the free wall of a ventricle. Although it is of unknown etiology, it is probably congenital. The weakness of the myocardial wall during embryogenesis can lead to a focal protrusion of the heart wall^(3)^. An LVD has a narrow neck through which it communicates with the ventricular cavity; in contrast, a left ventricular aneurysm (LVA) has a wide base for connecting with the ventricular cavity^(2)^. The wall of an LVA is akinetic, whereas an LVD contracts synchronously with the ventricle^(1,2)^. An LVD can be accompanied by other congenital and cardiac anomalies such as the pentalogy of Cantrell^(3)^.

The prenatal diagnosis of LVD or LVA can be made by ultrasound, and these anomalies are frequently accompanied by pericardial effusion, which can cause fetal pulmonary hypoplasia and progressive hydrops^(4)^. LVD is reported to have a more favorable long-term prognosis than does LVA^(5)^. The prognosis is usually favorable when there is no change in the size of the diverticulum, which was the case in the patient described here. When a fibrous LVD has a thin wall, disruption can occur and is usually fatal, although such a development is rare^(6)^. Prenatal monitoring, with serial examinations by fetal cardiology and cardiac surgery teams for proper programming of prenatal or postnatal interventions, will therefore be necessary^(7)^.
